# Considerations and cautions for the integration of psilocybin into routine clinical care: a consensus statement from the US National Network of Depression Centers' Task Group on Psychedelics and Related Compounds

**DOI:** 10.1016/j.eclinm.2025.103517

**Published:** 2025-09-23

**Authors:** Megan M. Hosein, Matthew J. Reid, Sarah Walser, Stuart Charney, Gregory A. Fonzo, Benjamin R. Lewis, David B. Yaden, Trisha Suppes, Zachary A. Cordner, Frederick S. Barrett

**Affiliations:** a Department of Psychiatry and Behavioral Sciences, Johns Hopkins University School of Medicine, Baltimore, MD, USA; b SixDegrees Health Care Consulting, Chicago, IL, USA; c Department of Psychiatry and Behavioral Sciences, Dell Medical School, Austin, TX, USA; d University of Utah Huntsman Mental Health Institute, Salt Lake City, UT, USA; e Department of Psychiatry and Behavioral Sciences, Stanford University, Palo Alto, CA, USA; f VA Palo Alto Health Care System, Palo Alto, CA, USA; g MindWork Group, Baltimore, MD, USA

**Keywords:** Psilocybin, Psychedelics, Consensus

## Abstract

The potential for psilocybin, and other psychedelic drugs, to fulfil a much needed and potentially transformative class of psychiatric treatments has garnered significant attention. Consequently, there has been a great deal of interest and investment in accelerating its development and potential implementation in routine clinical practice. However, the expanding scope of scientific discovery, heightened media coverage, and commercial interests in the field risk outpacing the rate of developments in the necessary guidelines and infrastructure required for integration of psilocybin into clinical practice. The US National Network of Depression Centers (NNDC) Task Group on Psychedelics and Related Compounds—comprising psychiatrists, psychologists, neuroscientists, psychedelic researchers, and leaders in healthcare consulting affiliated with the NNDC—developed this consensus statement as a summary of clinical expertise and opinion on the matter, to recognise psilocybin's therapeutic potential while also emphasising the need for further research and careful consideration before the integration of psilocybin into routine clinical care. We outline the current state of the science on psilocybin, incorporating articles published through April 2025. We identify key areas for further research and frame them within the context of therapeutic and ethical implications surrounding psilocybin's use in future clinical practice. We highlight the need for further research to address gaps in understanding of therapeutic dosage, efficacy across diverse populations, and long-term safety. Finally, we propose an agenda which calls for diversification of funding, collaborative research efforts, standardised training for healthcare providers, and careful consideration of ethical dilemmas inherent in the theorised clinical use of psilocybin. Crucially, we advocate for a balanced approach that prioritises rigorous scientific standards while considering the urgency of the need for better treatment options, ensuring equitable access and safety as the field progresses. We acknowledge that the single-country focus of the NNDC may limit the generalisability of recommendations to international contexts with differing healthcare systems and regulatory landscapes.

**Search strategy and selection criteria**

We conducted a Review of publicly available databases, including PubMed, Cochrane Library, and EMBASE databases, for relevant publications published between database inception and April 2025. The search was limited to articles published in English. The search terms used in PubMed and Cochrane Library included “psilocybin”, “psychedelics”, “major depressive disorder”, and “psychedelic assisted therapy”. We included various types of publications such as research studies, review articles, editorials, and clinical trials published until April 2025. We examined the secondary references of relevant papers to identify additional articles not captured by the electronic search. Articles were also identified through searches of the authors' own files. The final reference list was generated on the basis of originality and relevance to the broad scope of this Review.

## Introduction

In recent years, psychedelic medicines have surged to the forefront of public attention as a potential new class of much-needed psychiatric therapeutic agents. Research into the applications of psilocybin has been largely encouraging, further promoting broad hope for psilocybin's potential to provide relief for individuals suffering from some of our most difficult-to-treat conditions. With positive findings from the completion of multi-national Phase-II clinical trials with psilocybin for the treatment of depression, and with Phase-III registration trials underway, many are anticipating a future “post-regulatory era”. As members of the National Network of Depression Centers (NNDC), we share in this hope and recognise potential opportunities for breakthrough progress in the treatment of several psychiatric disorders if investment and public commitment to the development of psychedelic medicine continues. However, given the early stage of this field, many obstacles must first be addressed before we can arrive at this position. In this consensus statement, we discuss critical considerations as we move towards a future when psilocybin may be implemented into routine psychiatric care. In doing so, we outline areas of critical need for further investment and research, and we highlight several ethical considerations for the delivery of psychedelic therapy. Although other molecules such as DMT and LSD have been studied for depression and other indications, the bulk of contemporary evidence to date has arisen from the study of psilocybin, and as a result it is the only drug with reasonable prospects of clinical implementation for mood disorders in the near-term. For these reasons, we elect to limit the focus of this piece to psilocybin.

Although protocols have differed, most of the efficacy-determining studies to date have administered psilocybin alongside some degree of psychotherapeutic support. Typically, psychotherapeutic support is given before and after psilocybin administration (historically referred to as “psychedelic therapy”). This contrasts with the prevailing model used with MDMA, where psychotherapeutic support is given during administration (historically referred to as “psycholytic therapy”). Different terminology has been used in the literature to reflect these different interventions. We will broadly refer to psilocybin therapy as it has been delivered in the field to date, encompassing the diversity of dosing and psychotherapeutic protocols implemented in psilocybin research.

To date, a growing body of evidence has shown great promise for the use of psilocybin in the treatment of major depressive disorder (MDD),^1^, ^2^, ^3^ alcohol use disorder (AUD),^4^^,^^5^ and tobacco use disorder.^6^^,^^7^ Results from several Phase-II studies of psilocybin for depression have been replicated with positive and sustained results,^1^^,^^8^^,^^9^ which have led to ongoing Phase-III registration trials investigating its use for MDD^10^ and treatment-resistant depression.^11^^,^^12^ Many other investigations are also currently underway, including Phase-I or Phase-II trials evaluating psilocybin for the treatment of a range of other psychiatric, neurological, inflammatory, and other illnesses. While psilocybin remains a Schedule I substance and is approved by the FDA for investigational use, there are ongoing efforts at the state level to promote access and decriminalisation. For example, in 2020, Oregon became the first state to legalise “psilocybin services” at licenced centres, and in 2022 Colorado passed the Natural Medicine Health Act decriminalising psilocybin use along with several other psychedelic substances that exist in organic forms. In 2025, the New Mexico Medical Psilocybin Act established a regulated medical psilocybin program for licenced healthcare professionals to administer natural psilocybin for qualifying conditions in supervised settings.^13^ In addition, multiple countries, including Australia^14^ and Canada,^15^ have already granted early compassionate access to psilocybin, allowing it to be used clinically in limited cases, and such compassionate use has been ongoing in Switzerland for many years. Even so, the clinical application of psychedelics in the US, Canada, and Australia has been incredibly limited and represents only a very limited scope of practice as explicit therapy. Further, such use has not been accompanied by open and rigorous tracking of patient outcomes, limiting the utility of these examples for informing cautions and considerations for the integration of psilocybin into routine clinical care.

Studies at the molecular, cellular, and neurocircuitry levels are likely to inform our understanding of the mechanisms of psilocybin's therapeutic actions. For example, psilocybin has been shown to promote neuroplasticity via hippocampal neurogenesis,^16^ medial prefrontal cortex (PFC) dendritogenesis,^17^ and rapid induction of neuroplasticity-related gene expression in the PFC and hippocampus.^18^ In studies of patients with MDD, EEG correlates of increased neuroplasticity have also been observed following psilocybin-administration, relative to placebo.^19^ Further questionnaire evidence for increased psychological flexibility^20^ as well as behavioural evidence for increased cognitive flexibility and fMRI evidence for increased neural flexibility after treatment of depression^21^ are consistent with a neuroplastic therapeutic target of psilocybin. Psychedelic medicine practices that harness this neuroplastic change may prove to potentiate and increase the durability of therapeutic response. As these clinical and translational findings emerge, many are also preparing to equip health care workers with the knowledge and skills needed for psychedelics to translate into meaningful, patient-level clinical impact.^22^^,^^23^ Nevertheless, much work remains to better understand the utility of psychedelics, their long-term safety profiles in real-world clinical populations, their likely diverse biological mechanisms, appropriate training across the healthcare workforce, and best practices for practical and equitable clinical implementation. There are also substantial ethical considerations required for a field currently dominated by private and philanthropic funding, and a non-clinical market based around psychedelics for promotion of general wellbeing or spirituality. In particular, psilocybin administration often results in the evocation of intensely meaningful experiences as well as malleable mental states among potentially vulnerable individuals, and as such clearly requires a unique level of prudence and careful ethical considerations in its implementation. The following sections provide a detailed account of these considerations, summarised by calls to action and research recommendations.

The aims and objectives of this consensus statement are to evaluate the current scientific and clinical landscape of psilocybin as a potential psychiatric treatment, and to provide expert consensus on its responsible integration into clinical practice. This includes identifying research priorities, addressing ethical and therapeutic considerations, advocating for rigorous scientific standards, and promoting equitable access. There was no ethics committee approval for the writing of this manuscript. All participants of the taskforce provided informed consent to participate and are authors of this work.

The National Network of Depression Centers (NNDC) Task Group on Psychedelics and Related Compounds—comprising psychiatrists, psychologists, neuroscientists, psychedelic researchers, and leaders in healthcare consulting affiliated with the NNDC—developed this consensus statement as a summary of clinical expertise and opinion on the matter. The NNDC is a collaboration among 27 leading academic medical centres across the USA with 11 task groups dedicated to focused areas of interest. The Psychedelics and Related Compounds Task Group consists of approximately 50 members who are representatives of their member institutions with specific expertise in psychedelics. All task group members were invited to contribute to this paper on an opt-in basis, and further details on the composition of the taskforce are available in the Supplementary Materials. To outline the current state of the science on psilocybin, we searched for relevant articles published through April 2025. Further details can be found in the Search Strategy and Selection Criteria panel.

## Addressing the current state of the scientific literature

Investigations into the effects of psilocybin began to re-emerge in the early 2000s. In 2006, Griffiths et al. published a now widely cited paper demonstrating the potential of psilocybin to occasion mystical experiences in 36 healthy individuals.^24^ Along with revealing the potential for deeply meaningful and positive experiences following psilocybin dosing, this study also indicated the potential safety and feasibility of using psilocybin in future studies. A smaller confirmatory study (n = 18) soon followed exploring neurocognitive effects of psilocybin and its safety profile across a range of doses, from 0 to 30 mg/70 kg psilocybin.^25^ In line with these two studies, the first therapeutic study was also published in 2006, and demonstrated an acute reduction of symptom severity in 9 patients with obsessive-compulsive disorder (OCD).^26^ The next therapeutic study soon followed, investigating psilocybin as an open-label intervention for depressive and anxiety symptoms in 12 patients suffering from advanced-stage cancer.^27^ Subsequently, two larger double-blind placebo-controlled trials replicated these findings, demonstrating a robust and sustained therapeutic effect of psilocybin in patients with cancer.^28^^,^^29^ The first cohort of 51 participants demonstrated a significant improvement in depressive symptoms (HAM-D: 89% response, 71% remission) and anxiety symptoms (HAM-A: 80% response, 50% remission) at 6 month follow up.^28^ In the second study of 24 participants, there were both anxiolytic (78% response) and antidepressant (58% response) effects relative to control (14% for both antidepressant and anxiolytic response) at 7 weeks which was sustained at both 6·5 months,^29^ and 4·5 years follow-ups.^30^

Since then, additional early-phase studies have been initiated to test psilocybin as a potential treatment for a wide variety of neuropsychiatric, neurological, and other disorders. Currently MDD remains the most extensively studied. An open label pilot study of 12 individuals with treatment-resistant depression showed a response rate of 67% one week after treatment, and 42% remaining in remission at 3 month follow up.^31^ A later wait-list controlled trial observed a clinical response of 71% and remission in 54% of 24 patients four weeks after two doses of psilocybin (20 mg/70 kg followed by 30 mg/70 kg),^1^ with a sustained response at 12 month follow up.^8^ Other recently published studies include a large phase-2 double-blind randomised controlled trial (RCT) investigating psilocybin plus psychological support during administration for treatment-resistant depression, which showed a positive yet more modest response of shorter duration compared to prior studies.^2^ Of the 79 patients in the high-dose (25 mg) treatment arm, 23 showed remission, which represented a higher remission rate than the 1 mg control condition (29% vs. 14% remission) over three weeks.^2^ However, the 10 mg group (low-dose) showed no significant difference compared with the control (1 mg) condition.^2^ Furthermore, suicidality was raised as a serious adverse event in both the 25 mg and 10 mg arms, prompting concern. Follow up phase III trials are ongoing, using the same dosing protocol along with a separate protocol that includes a second dose at three weeks. Another recent RCT of 104 participants with MDD comparing psilocybin + psychotherapy to niacin, placebo + psychotherapy showed a clinically significant response in 42% of the psilocybin group vs. 11% in placebo, and 25% remission in the psilocybin group vs. 9% in placebo.^3^ Although some of these response and remission rates are indeed striking, when viewed through the lens of non-response the results are more sobering. In these studies, 11–58% (mean 35%) of participants did not achieve treatment response, and 29–85% (mean 59%) did not achieve remission. Future work should consider precision medicine approaches to identify suitable candidates for therapeutic psilocybin while also exploring explanations of why some participants do not respond.

A recent direct comparison of psilocybin to escitalopram in the treatment of MDD has demonstrated that psilocybin did not significantly outperform escitalopram,^9^ when assessed using the primary outcome for depression (QIDS). In this study, from baseline to 6 weeks the psilocybin group had a reported mean change in QIDS of −8·0 ± 1·0 vs. −6·0 ± 1·0 in the escitalopram group. However, secondary outcome assessments of other clinically validated assessment scales mostly favoured psilocybin over escitalopram, suggesting that psychometrics and symptom characterisation may be critical in the evaluation of therapeutic response in patients with depression. Apart from MDD, psilocybin has also shown safety and potential utility in the treatment of AUD,^4^^,^^5^ tobacco use disorder,^6^^,^^7^ OCD,^26^ migraine headaches,^32^ cluster headaches,^33^ bipolar depression,^34^ and anorexia nervosa.^35^ Many other studies are ongoing, including investigations of psilocybin in comorbid MDD and AUD,^36^ chronic Lyme disease,^37^ depression and anxiety in Parkinson's disease,^38^ chronic suicidal ideation,^39^ fear of breast and ovarian cancer recurrence,^40^ phantom-limb pain,^41^ chronic low back pain,^42^ and depression comorbid with early Alzheimer's disease^43^ and mild cognitive impairment,^43^ among others. Research is also underway to investigate the effect of different dosing^44^ or psychotherapy protocols,^45^, ^46^, ^47^, ^48^ and the possibility of accessing the antidepressant effects of psilocybin without the psychedelic experience (e.g., via co-administration with a serotonin 5HT2A receptor antagonist such as risperidone).^49^

Overall, evidence for therapeutic benefits of psilocybin suggest promise, though several limitations should be acknowledged. Clinical trials, for example, are highly protocolised and controlled, and may not always generalise to non-research clinical settings. Investigations of diverse populations which adequately represent the target population of the disorders in question are much needed. For instance, more than 80% of the study participants in psychedelic trials have been white individuals^50^ which severely limits our ability to understand efficacy amongst other racial and ethnic groups, and in other underserved and underrepresented populations. Finally, therapeutic mechanisms of action are also understudied, and they are likely more complicated than first envisaged and may differ drastically across indications. In particular, the differential contributions of pharmacology and psychotherapy are closely intertwined and not yet separable based on the current literature, but psychotherapeutic approaches, whether structured or unstructured, could plausibly play a role in observed efficacy. Overall, therapeutic mechanisms likely involve diverse pathophysiological processes across cellular, circuit-based, cognitive, psychological, social and anthropological systems, requiring expansion of resources to new disciplines and multi-disciplinary collaboration to identify the contribution of any given process, level, or system to both risks and potential benefits of psychedelic therapies.

### Call to action

**Fig. 1:**
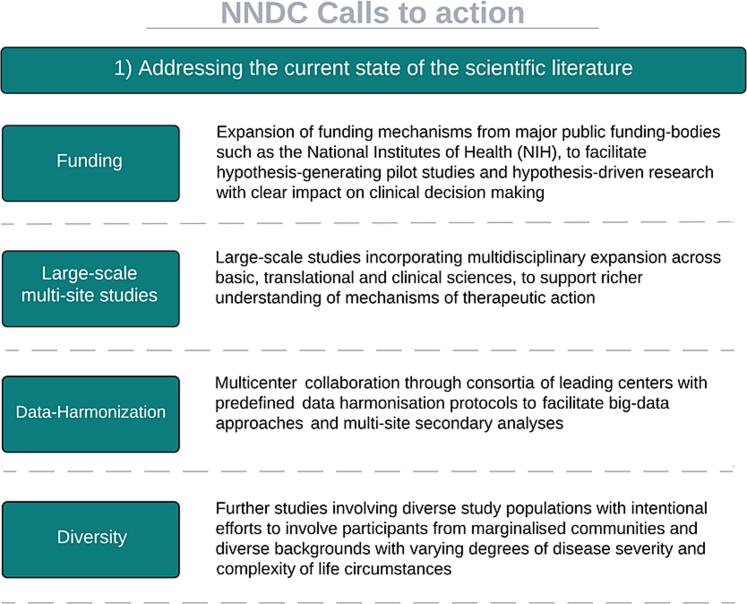
**NNDC call to action: addressing the current state of the scientific literature**.

To this end, we advocate for (Fig. 1):

1. Expansion of funding mechanisms, including those from major public funding bodies such as the National Institutes of Health (NIH), to facilitate rigorous and hypothesis driven evidence with clear impact on clinical decision making and to facilitate hypothesis generating pilot studies.
2. Large-scale studies incorporating multidisciplinary expansion across basic, translational, and clinical sciences, to support richer understanding of mechanisms of therapeutic action.
3. Multicentre collaboration through consortia of leading centres with predefined data harmonisation protocols to facilitate big-data approaches and multi-site secondary analyses.
4. Further studies involving diverse study populations with intentional efforts to involve participants from marginalised communities and diverse backgrounds with varying degrees of disease severity and complexity of life circumstances.
5. More robust longitudinal evaluations of potential harms of short-term and long-term psilocybin administration as well as potential risk factors for adverse events.
6. Large-scale factorial design studies to evaluate the relative contributions of drug vs. associated therapy as well as the interactions between drug and therapy.

## Clinical and practical considerations

### Safety

While most of the early studies of psilocybin have brought relative reassurance in terms of safety profile, limited representation in existing data clearly demonstrates a need for studies designed to include more diverse populations. Many unanswered questions include the potential for drug-drug interactions, optimal environment (‘setting’) and expectancy (‘set’) of participants completing dosing, the role of adjunct psychotherapy, longitudinal and sustained benefits, possible harms that have not surfaced in shorter duration trials, and considerations for its use in patients with major medical comorbidities.

Additionally, even in the existing limited literature, some complexities and potential safety concerns have emerged. Long acknowledged risks include the possibility of distressing or psychologically challenging experiences, heightened anxiety, or emergence of psychotic symptoms during the psychedelic experience.^51^, ^52^, ^53^ As clinical studies have grown in size and complexity, a possible signal for increased suicidal thinking and behaviour has emerged. For example, a recent phase-II trial of psilocybin for treatment-resistant depression reported serious adverse events in 9% of the 25 mg group (two participants with suicidal ideation, two participants with intentional self-injury), 7% in the 10 mg group (two with suicidal ideation, one with ‘intentional self-injury’, one hospitalisation for severe depression), and one in the placebo group (‘intentional self-injury’).^2^ While this study was too small to draw a link between suicidal ideation and psilocybin exposure, the findings do warrant careful consideration in future studies. Expectations about the psychedelic experience and disappointment at non-response or an underwhelming experience may feed into these adverse events - including one documented case of death by suicide after a patient with late-stage cancer received a placebo dose in Griffiths et al. (2016).^24^ Whether or not these considerations are more pertinent within psychedelic research than in other experimental therapeutics, evidence to date demands careful management and monitoring of expectations amongst vulnerable patients.

A number of other potential risks have been increasingly discussed but remain understudied. For example, the extent to which psilocybin, when used as a treatment for depression, may increase the risk of unmasking a bipolar spectrum disorder by triggering a mixed or manic episode is not well known. Case reports have demonstrated the validity of this concern.^54^^,^^55^ While recent open-label pilot studies in a small sample of participants with bipolar type II disorder have provided tentative evidence of psilocybin's safety in this population, no studies have been conducted for patients with bipolar type I disorder.^34^ It is not uncommon for those with bipolar disorder to be initially misdiagnosed with MDD, as depressive episodes are often frequent and occur earlier in the course of illness.^56^^,^^57^ While psychiatrists have experience and understanding of what a mood state transition may look like with typical antidepressants, it is still unknown how this may present after exposure to psilocybin and whether a mixed or manic episode triggered by psilocybin would respond in the same manner to currently available treatments. There are also concerns regarding Hallucinogen Persisting Perceptual Disorder (HPPD) occurring at a putative prevalence of ∼4% amongst recreational users.^58^ A prospective naturalistic survey of 654 individuals planning to take psychedelics (LSD 54% and psilocybin 41%) found that 30% of individuals at week 4 reported persistent perceptual symptoms (e.g., intensified colours and positive afterimages), but only 1% found it distressing, a necessary criterion for the diagnosis of HPPD.^59^ HPPD also has yet to be documented in clinical-trials, and large scale studies are needed to identify potential biomarkers, prodromal syndrome, and predictors of HPPD. The risk of long-lasting distressing or impairing perceptual disturbances or the unmasking of a psychotic illness by psilocybin warrants further study. Notably, a recent systematic review and meta-analysis of 214 unique studies found that classic psychedelics (e.g., LSD and psilocybin) were generally well tolerated in contemporary clinical and research settings, with serious adverse events reported in only 4% of participants with preexisting neuropsychiatric conditions.^60^ These adverse events included worsening depression, suicidal behaviour, psychosis, and convulsive episodes; there were no reports of death by suicide, persistent psychotic disorders, or HPPD following administration of high-dose classic psychedelics.

As current treatment protocols are conducted under the highly protocolised conditions required for research, the research studies to date ultimately lack generalisability to the level of environment in a routine clinical setting. Patients involved in research are rigorously screened and must first demonstrate a lack of exclusionary criteria or potential contraindications (e.g., history of bipolar disorder, psychosis or substance use disorder). In a true clinical environment, patient histories will likely be more complicated by partial or unclear information and determination of risk stratification will be more nuanced. Moreover, highly trained mental health care specialists are required to be present before, during, and after treatment sessions, and this poses staffing and reimbursement challenges in a standard clinical setting.

Beyond potential psychiatric complications, there remain hypothetical concerns about the effect of psilocybin's 5HT2B receptor agonists properties and valvular heart disease. The association of serotonergic drugs fenfluramine (phen/fen) and dexfenfluramine with valvular heart disease led to their withdrawal from the market in 1997,^61^ and some studies have linked repeated MDMA use with cardiac valvular disease,^62^ perhaps due to their effect of 5HT2B receptor stimulation in cardiac interstitial cells. With no data available on psilocybin, it remains to be seen whether the same phenomenon applies to psilocybin and other psychedelics and whether the risk is associated only with chronic repeated use.

### Drug-drug interactions that may limit effectiveness

Increasing attention has also been paid to potential drug-drug interactions. For example, concurrent selective serotonin reuptake inhibitor (SSRI) use has been thought to potentially diminish the effects of psilocybin.^63^ However current evidence is mixed, and ongoing studies are actively investigating this interaction. Becker et al. examined the acute effects of psilocybin following two weeks of escitalopram pretreatment and found no reduction in positive mood but a significant decrease in anxiety compared to placebo.^64^ Similarly, Goodwin et al. evaluated psilocybin treatment in 19 patients on chronic SSRIs and reported a 42% reduction in depression severity at week 3, suggesting that ongoing SSRI use may not hinder psilocybin's therapeutic potential.^65^ As several other antidepressants, neuroleptics, and non-psychiatric drugs exert effects on the serotonergic system, a broader investigation of this phenomena is likely warranted. If, in fact, SSRIs or other serotonergic medications limit the effectiveness of psychedelics to a substantial degree, significant limitations may arise in routine clinical care where washouts of medications may not be practical or safe. A critical safety concern regarding psilocybin in clinical practice involves this potential need to taper or discontinue some medications prior to psilocybin treatment. For some patients, tapering or discontinuing certain medications in pursuit of treatment with psilocybin may not be feasible. When attempted, this process should be carried out in a safe, well-supervised setting by a provider with the expertise to navigate the risks and benefits of medication tapering, as it can be challenging and may significantly increase the risk of worsening depression. Further evidence suggests that the diminished therapeutic effect caused by previous SSRI/SNRI use may ultimately be un-addressable, even after discontinuation prior to psilocybin dosing.^66^ In addition to these interactions that may limit effectiveness, at least one recent study identified a potentially hazardous drug-drug interaction. In a naturalistic review of internet self-reports of psilocybin self-administration, Nayak et al. found that psilocybin co-administration with lithium, but not lamotrigine, may increase seizure risk.^67^

### Uncertainty about most effective dosage

Aside from adverse effects, there is also still relatively little known about optimal dosage to achieve desired therapeutic effects. While a canonical dose of 25 mg has driven therapeutic response in the most recent clinical trials, it is unclear whether this is truly the optimal dose. Likewise, it remains unclear if different indications may maximally benefit from different dosages. Evidence may suggest a higher dose (up to 40 mg of psilocybin) may be necessary in patients with AUD.^5^ In addition to questions of drug dosage, similar questions remain regarding the necessity of and best practices for integration of psychotherapy within psilocybin treatment.^68^

### Questions about protocol, health economics, scalability, and accessibility

Related to dosage and the combination of psilocybin with therapy, several treatment protocols have been manualised for study purposes.^69^^,^^70^ While this allows for more rigorous study and replication, most treatment protocols remain resource-intensive, and there is limited data on what the critical elements contributing to safety and effectiveness are within each protocol. Currently the ability to generalise protocols across indications also remains unclear. If the clinical benefits turn out to require implementation of something resembling the full versions of protocols currently in common use, barriers may emerge to scalable, affordable, and equitable access in routine clinical settings. A recent study estimated that the cost of psilocybin treatment combined with therapy from one therapist would be £5239 (∼$6600) if psilocybin is costed at £1200 (∼$1500),^71^ and psilocybin was only determined to be cost-effective compared to other modalities when the cost of therapist support was reduced by 50% and the cost of psilocybin was reduced to £400 (∼$520) per person. In Australia, private insurers have created “proof of concept” psychedelic therapy clinics and are actively evaluating the health economics to assess whether to include these treatments.^72^ Additionally, while some have worked to develop roadmaps for coding and billing,^73^ many practical questions remain, and self-pay models are already being developed, which could adversely impact equitable access.

### Call to action

**Fig. 2:**
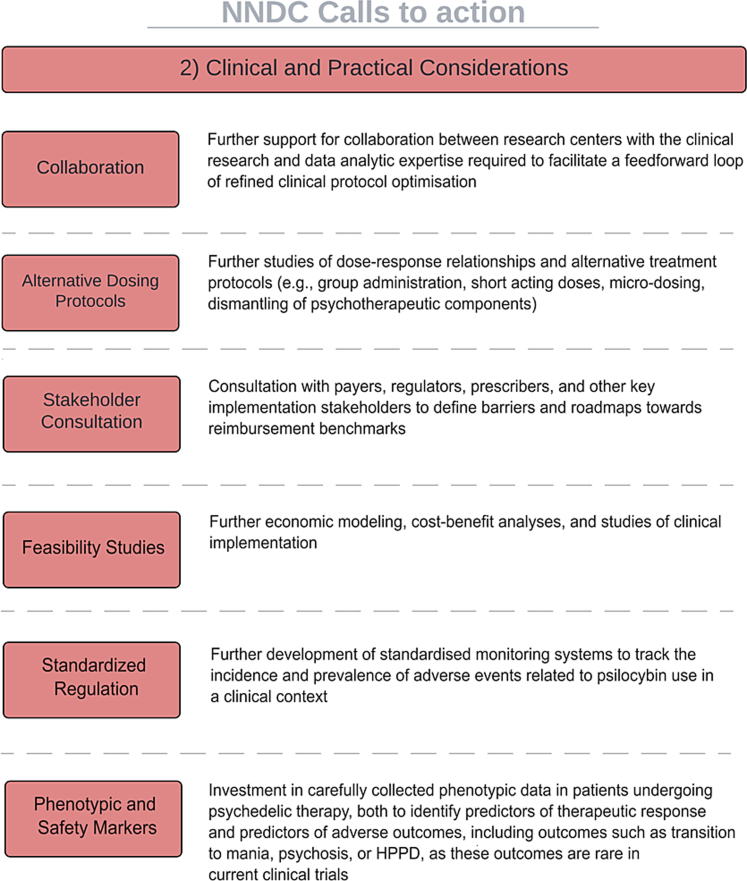
**NNDC call to action: clinical and practical considerations**.

We recommend (Fig. 2):

1. Further support for collaboration between research centres with the clinical research and data analytic expertise required to facilitate a feedforward loop of refinement of clinical protocol optimisation.
2. Further studies of dose-response relationships and alternative treatment protocols (e.g., group administration, short-acting doses, micro-dosing, dismantling of psychotherapeutic components).
3. Further development of standardised monitoring systems to track the incidence and prevalence of adverse events related to psilocybin use in a clinical context.
4. Investment in carefully collected phenotypic data in patients undergoing psychedelic therapy, both to identify predictors of therapeutic response and predictors of adverse outcomes, including outcomes such as transition to mania, psychosis, or HPPD, as these outcomes appear rare and elusive in current clinical trials data.
5. Further economic modelling, cost-benefit analyses, and studies of clinical implementation.
6. Consultation with payers, regulators, clinician, and other key implementation stakeholders to define barriers and roadmaps towards reimbursement benchmarks.

## Education and training opportunities

Currently, standardised evaluation of provider competence is nonexistent. Development of formal curricula and requirements is needed and to date remains in its infancy, largely due to a dearth of requisite data to guide a framework. Many vital questions remain unanswered, such as what will the American Board of Psychiatry and Neurology (ABPN) requirements be for residents in-training, or continued medical education (CME) requirements for psychiatrists, psychotherapists/guides, pharmacists, social-work, nursing, and other clinical colleagues. Stakeholder consultation may prove beneficial at this early stage and should be initiated in anticipation of potential future regulator approvals. There remains an important conversation surrounding whether there should be a required gatekeeper program enforced (similar to Risk Evaluation and Mitigation Strategies [REMS] programs for monitored medications such as clozapine). For many medical specialities, training regularly requires that procedures and competencies be verified during residency—should we be establishing the same for psychiatrists and other providers who may be on the cusp of delivering these new treatment modalities?

Any efforts to provide safe, rational, and broad access to psilocybin treatment will require well-informed providers. From a practical perspective, there are obvious challenges for the development of evidence-based educational programs. The urgency of acknowledging these challenges is made greater by the current rush towards liberalising access before well-informed training is available for health care providers or first responders. Currently, training opportunities primarily exist through apprenticeship-like models and several retreat-like programs in a variety of non-standardised, unaccredited settings, where details vary widely, data on outcomes are lacking, and their design may be explicitly experiential. In addition, there is no standardisation or current evidence base to validate these programs' curricula. While offering such opportunities may improve access to treatment, this also risks creating an environment without rigour or oversight.

Given the lack of evidence-based training curricula, due to a lack of evidence-base, we risk entering hazardous terrain wherein researchers are moving ahead with evidence on therapeutic efficacy of these treatments, while the training for broad clinical use is not at all ready. Consequently, many questions await answers through more clinical research, such as what level of training is necessary, and what are the short- and long-term safety risks for prescribers to understand. Encouragingly, multisite efforts are underway to develop a formalised curriculum, including one in its infancy currently being studied and evaluated in a psilocybin training collaborative between Yale, New York University and Johns Hopkins Hospital. The ideal training program would harmonise between the many fields involved—pharmacy, psychology, nursing, social work, as well as physicians and alternative prescribers (e.g., nurse practitioners and physician assistants).

### Call to action

**Fig. 3:**
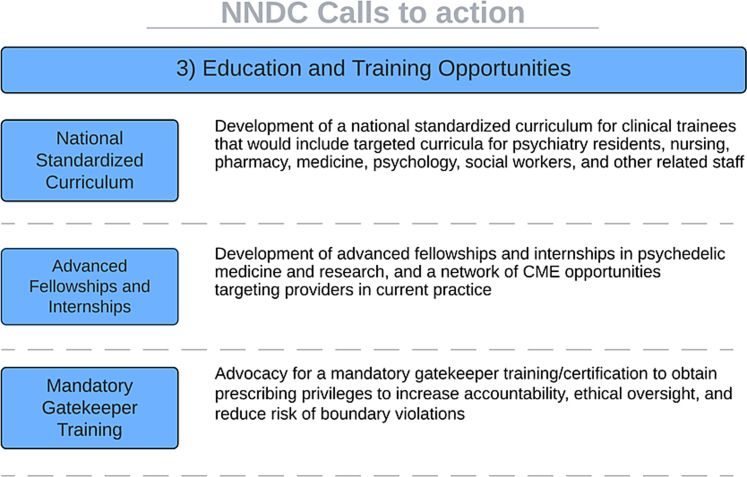
**NNDC call to action: education and training opportunities**.

To address these critical gaps, we recommend that the relevant stakeholders begin to initiate the following processes (Fig. 3), in anticipation of the long-term potential of psilocybin approval:

1. Development of a national standardised curriculum for clinical trainees that would include targeted curricula for psychiatry residents, nursing, pharmacy, medicine, psychology, social workers, and other related staff.
2. Development of advanced fellowships and internships in psychedelic medicine and research, and a network of CME opportunities targeting providers in current practice.
3. Advocacy for a mandatory gatekeeper training/certification to obtain prescribing privileges to increase accountability, ethical oversight, and reduce the risk of boundary violations.

## Ethical considerations

The medical implementation of psilocybin poses a number of unique and important ethical considerations with respect to regulation, access, informed consent, and scope of practice. The field of psychiatry has a duty to remain critical and hold emerging interventions to a scientifically rigorous standard, while also balancing this with great unmet need and therapeutic potential. Much of the current debate is also influenced by the psychedelic movement of the 1950s and 1960s which was fraught with ethical and institutional boundary violations. Current public perception is overall positive with 61% of Americans supporting regulated therapeutic use of psychedelics.^74^ However, public enthusiasm is also countered by institutional alarmism, with influence from decades of the War on Drugs messaging, and both forces must be acknowledged with equanimity.

This becomes particularly challenging when faced with the methodological complexities of study design, participant selection, and blinding in psychedelic clinical trials.^75^ In the case that some researchers have had their own personal experiences with psychedelics, they may bring unwarranted enthusiasm that may introduce multiple biases.^76^ Personal experience and investment in a research topic is not unique to the psychedelic arena and is not necessarily a shortcoming, but there is a dearth of understanding of how such experience could frame their use and influence outcomes, both good and bad.

In addition to these methodological considerations, there are also external pressures of public opinion, speculation on potentially lucrative markets, academic ambition, and expanding public access in response to pockets of decriminalisation. Even with a growing body of scientific evidence, it is difficult to predict how highly controlled experiences, facilitated by trained therapists in clinical trials will translate into the public arena. Just and equitable access to this therapy will also be a challenge, as the current model is time intensive and may not be uniformly covered by insurance, thus potentially worsening already existing healthcare disparities.

Psychedelics also require a reassessment of the traditional informed consent process in medicine. Due to the unique vulnerabilities experienced under the effect of psilocybin, a more thorough educational and preparatory phase may be required to facilitate a deeper understanding of the risks and benefits of this experience.^77^ The medical ethics of protection must be clarified in the context of the psilocybin experience itself, due to its capacity to alter cognition, openness, and trust. Participants may be more open to suggestion. Boundary violations in this setting may occur on the part of an unscrupulous practitioner, but also on the part of the patient as an effect of their altered state. Third party monitoring or videorecording could help to protect patients from provider transgressions. Proper training of both patient and practitioner on how to handle blurred boundaries could help those involved effectively navigate these situations. In addition to clear egregious violations like sexual contact, the psychedelic space should also be mindful of ideological transgressions such as the invocation of practitioner derived religious and non-empirically based theory in a clinical setting.^78^

A core tenet of psychiatry is the commitment to caring for those experiencing mental distress and illness. This raises questions about the role medicine should have in governing how psilocybin is used, particularly when it has been used in non-medicalised settings for centuries (i.e., among certain indigenous groups) for its spiritual and consciousness altering potential in people who are not experiencing a mental illness. The medicalisation and commodification of the psilocybin experience, if not executed mindfully, also threatens to extract aspects of traditional indigenous knowledge, practices, and heritage without just and respectful reciprocity.^79^

### Our recommendations: clinical and public health implications

These recommendations carry the potential for broad clinical and public health implications, through guiding policy development, supporting equitable access, and helping prevent premature or unsafe implementation as interest in psychedelic therapies grows. Clinicians and the public should approach psychedelics with optimism but caution, as psychedelics are neither widely approved nor clearly understood within a health system context. Messaging regarding the status and promise of psychedelic medicine should be tempered by these facts. In addition, more preparation and education of lawmakers, policymakers, care providers, and the public is needed regarding the potential risks that are known (e.g., in those with potential cardiovascular vulnerabilities), suspected (e.g., unmasking mania and precipitating psychosis in those with specific psychiatric vulnerabilities), and as of yet unknown (e.g., 1/1000 or 1/10,000 risks that have yet to be encountered in clinical trials). Careful monitoring of psychedelic use and outcomes in both clinical and unsanctioned settings will aid in tracking and responding to these risks as access to psychedelics increases. Care must be taken to ensure that patients are adequately protected in periods of immense psychological vulnerability that can be encountered with psychedelic therapy. Given all of the known and unknown risks as well as our scope of knowledge regarding efficacy, psychedelics might best be considered as second- or third-line therapies until we gain more clarity about the use of psychedelics in the clinic. An adequate evidence base must be developed to guide the appropriate level of training and expertise that is necessary to ensure the safety of patients undergoing psychedelic therapy. Finally, the limits of efficacy of psychedelic therapy must be more carefully charted and communicated. These efforts will require a substantial commitment to monitoring, rigorous implementation science, and continuing education to best ensure a safe roll-out of psychedelics, if they are approved as therapies.

### Our recommendations: caveats

While bringing together a diverse group of U.S.-based experts, the single-country focus of the NNDC may limit the generalisability of recommendations to international contexts with differing healthcare systems and regulatory landscapes. Additionally, while the group achieved consensus through a process of collaborative discussion and iterative drafting, it did not include a formal Delphi methodology. Such an approach may enhance and strengthen such recommendations in the case that a more broad and inclusive set of considerations and opinions are generated in future work. Further, the inclusion of patients with lived experience with psychedelic therapy may be useful in the future, but at this time the available lived experience is restricted to patients enrolled in clinical trials as well as individuals undergoing psychedelic therapy in unsanctioned settings. Both these factors relate in an unclear manner to the way in which psychedelics may be delivered in a clinical care setting after FDA approval, and thus might limit the generalisability of lived experience to future clinical settings. Future reviews and perspectives will be well-informed by experience in a post-approval world.

## Conclusions

We see promise in the current wave of research in psychedelic medicine. At the same time, as physicians and medical researchers, we encourage caution given unanswered questions and practical implementation challenges that do not yet have clear, generalisable solutions. We highlight the need for the development of standardised training across a broad swath of disciplines in the event that this new field of medicine becomes a potential tool in our therapeutic toolbox. We emphasise the need to maintain guiding principles of medical ethics, even if doing so involves taking unpopular positions in the face of public and commercial enthusiasm. We believe strongly in the need for more research on psychedelics in the context of mental health, with special attention to their potential risks, and more multi-site collaboration and harmonisation to work towards answering some of these most pressing questions.

## Contributors

MH: Conceptualisation, literature review, writing—original draft, review & editing. MR: Visualisation, writing—review & editing. SW: Conceptualisation, writing—review & editing. SC, GF, BL, DY, and TS: Writing—review & editing.

ZC: Conceptualisation, writing—original draft, review & editing; responsible for overseeing the paper and ensuring the integrity of the research process; and provided guidance throughout the writing of the paper. FB: Supervision, writing—review & editing, project administration; provided guidance throughout the writing of the paper and ensured the accuracy of the final submission.

All authors have read and approved the final manuscript and agree to be accountable for all aspects of the work.

## Declaration of interests

BL has pending support from NIDA for a randomised controlled trial related to ketamine and opioid use disorder, and funding from NIH/NCI for a study on psychedelic-assisted psychotherapy and mindfulness training. He has received NIH/HCI funding for a study on prostate-cancer related fatigue. He has received industry funding from Reunion Neuroscience for a trial of a novel short acting psychedelic for women with post-partum depression, and from COMPASS Pathways for a Phase III trial of COMP360 in participants with treatment resistant depression. GF received investigational drug for an ongoing study of psilocybin for treatment-resistant depression from COMPASS Pathways. He has received consulting fees from Synapse Bio AI and payments for lectures from the American Association of Psychiatric Pharmacists, Texas Research Society for Alcoholism, and University of Texas Psychiatric Pharmacotherapy Update. He has also received grants from One Mind—Baszucki Brain Research Fund, the SEAL Future Foundation, and Brain and Behavior Research Foundation. He is the Chair of the Research Track for the Alies-Muskin Career Development and Leadership Program of the Anxiety and Depression Association of America. DY has received research funding to his institution from the National Institute of Health NCCIH, Heffter Research Institute, Steven and Alexandra Cohen Foundation, and Templeton World Charity Foundation. TS has received grants or contracts from Compass Pathways, Merck, National Institute of Health, VA Cooperative Studies Program, VA ORD Prime Care, National Institute of Drug Abuse; she has received royalties or licences from the American Psychiatric Association of Publishing, Hogrege Publishing, Jones and Bartlett, Wolters Kluwer Health (UpToDate), MindMed, Servier (Australia), Allergan, Inc. She has received payments or honoraria from Medscape (WebMD), Intracellular Therapies, CMEology, Novus Medical Education, CME Institute (Physicians Postgraduate Press, Inc). MR has received grants from Source Research Foundation and the American Academy of Sleep Medicine unrelated to this manuscript; he has received support and patents for work unrelated to this manuscript. FB received study drug (psilocybin) from Usona Institute and Purisys, Inc. for a clinical trial not reported in this manuscript. ZC, SW, SC, MH have no conflicts of interest.

## Appendix A Supplementary data

NNDC Task Group Members
