# Supplementary material for: Considerations and cautions for the integration of psilocybin into routine clinical care: a consensus statement from the US National Network of Depression Centers' Task Group on Psychedelics and Related Compounds
Source: eClinicalMedicine. 2025 Sep 23;89:103517. doi: 10.1016/j.eclinm.2025.103517 (PMC12495261; doi:10.1016/j.eclinm.2025.103517)
Supplement: NNDC Task Group Members [file mmc1.docx]

| **Surname** | **First name** | **Country** | **Profession/discipline** | **Years of experience** | **Role in group** |
| --- | --- | --- | --- | --- | --- |
| Hosein | Megan | USA | Psychiatry, Internal Medicine | 9 | Conceptualization, selecting and reviewing evidence, writing and editing manuscript |
| Reid | Matthew | USA | Neuroscientist | 10 | Visualization, reviewing and editing manuscript |
| Walser | Sarah | USA | Psychiatrist | 3 | Conceptualization, writing and editing manuscript |
| Charney | Stuart | USA | Attorney, Health Policy expert | 39 | Co-chair NNDC Advisory Council, reviewing and editing manuscript |
| Fonzo | Gregory | USA | Clinical psychology | 10 | Reviewing and editing manuscript |
| Lewis | Benjamin | USA | Psychiatrist | 13 | Reviewing and editing manuscript |
| Yaden | David | USA | Experimental psychology | 10 | Reviewing and editing manuscript |
| Suppes | Trisha | USA | Psychiatry, Clinical Trials in  Mood and Anxiety Disorders | 35 | Co-chair of Task Group, reviewing and editing manuscript, formulation of recommendations |
| Cordner | Zachary | USA | Psychiatry and Behavioural Neuroscience | 9 | Conceptualization, writing and editing manuscript, formulation of recommendations |
| Barrett | Fred | USA | Cognitive Neuroscience  and Behavioural Pharmacology | 12 | Co-chair of Task Group, supervision, reviewing and editing manuscript, project administration, formulation of recommendations |
